# Overt Acute Hepatitis B Deteriorates in Females: Destructive Immunity With an Exaggerated Interleukin-17 Pathway

**DOI:** 10.3389/fimmu.2021.631976

**Published:** 2021-11-11

**Authors:** Ming-Ling Chang, Chau-Ting Yeh, Rong-Nan Chien, Yun-Fan Liaw

**Affiliations:** ^1^ Liver Research Center, Division of Hepatology, Department of Gastroenterology and Hepatology, Chang Gung Memorial Hospital, Taoyuan, Taiwan; ^2^ Department of Medicine, College of Medicine, Chang Gung University, Taoyuan, Taiwan

**Keywords:** female, AHB, IFN-γ, IL-4, IL-17

## Abstract

**Background and Aims:**

We previously showed that overt acute hepatitis B (AHB) was more severe in female patients. Using the same cohort and AHB mouse model, we examined the underlying mechanism.

**Methods:**

Baseline biochemistry, virological and cytokine assays, and T helper (Th)1 and Th2 immune markers of 118 consecutive patients were analyzed. The decompensated livers of AHB and chronic hepatitis B (CHB) patients who underwent liver transplantation were analyzed immunohistochemically. B6 mice were hydrodynamically injected with pHBV1.3 plasmids.

**Results:**

Decompensated AHB patients (n=41) were older, more often female, and had higher alanine aminotransferase (ALT), soluble programmed cell death protein 1 (sPD-1) levels, and neutrophil-lymphocyte ratios but lower rates of HBeAg positivity and quantitative HBsAg, interferon (IFN)-γ-inducible protein 10 (IP-10), IFN-γ, and interleukin-4 (IL-4) levels than the compensated patients. Female sex (95% CI OR=1.07~54.9), age (1.06~1.40), and ALT levels (1.001~1.004) were associated with hepatic decompensation. Higher sPD-1 but lower IFN-γ and IL-4 levels were observed in female patients. Compared to CHB, decompensated AHB livers had more IL-17-positive cells but fewer HBsAg-positive cells and lower CD4/CD8 ratios. Higher serum IL-17 levels were noted in the female AHB mice than those in the males.

**Conclusions:**

Females predominated in decompensated AHB, in which downregulated IFN-γ and IL-4 with augmented hepatic IL-17-positive cell development indicated accelerating destructive immunity to enhance viral clearance. The early surge of serum IL-17 was confirmed in the female AHB mice. Targeting the pathway involving IFN-γ, IL-4, and IL-17 might prevent liver transplantation or fatality in decompensated AHB.

## Introduction

Although the global incidence of acute hepatitis B (AHB) is as low as 2.33/10^5^ because of the wide application of the universal hepatitis B virus (HBV) vaccination (1), the rate has recently increased, particularly among high-risk populations exposed to unsafe sexual activity and intravenous drug use (2). HBV exposure leads to AHB, which is usually resolved within 6 months in approximately 95% of adults; however, the viral infection is not controlled in the remaining 5%, leading to chronic hepatitis B (CHB) (3). Moreover, approximately 1% of AHB cases manifest as severe or fulminant hepatitis characterized by a fatality rate of up to 80% (4). It is not unusual that severe or fulminant AHB cases rely on liver transplantation as a life-saving intervention (5). Although the advent of potent nucleos(t)ide analogues (Nucs) may rescue most severe hepatitis flares associated with CHB (6), their efficacy in preventing severe or fulminant AHB is not as high as that for CHB. Knowing the factors and associated factors determining the prognosis of AHB is crucial to prevent severe AHB prognoses. Various host and viral factors affect the progression of infectious liver disease. The viral load of AHB is significantly lower than that of CHB (7), and certain fatal AHB cases have even demonstrated an undetectable HBV viral load (8). Thus, the role that Nucs play in treating severe AHB remains controversial (9, 10). Furthermore, in contrast to the finding that acute exacerbation in CHB is usually associated with more complications in male patients (11, 12), our recent study showed that overt AHB is more severe in female patients, despite their lower viral load compared to men (13). Therefore, host factors, particularly sex-specific ones, are likely more important than viral factors in determining AHB prognosis. Accordingly, the current study used the same patient cohort to determine the biological basis of AHB decompensation, with a particular emphasis on host factors. Additionally, immunohistochemical (IHC) analyses were used to examine the decompensated livers from AHB patients compared to those from CHB patients. In parallel, in AHB mouse models, the underlying mechanism was verified through hydrodynamic injection with HBV plasmids.

## Methods

### Patients

As previously described, the study consisted of adults (18 yrs and older) with overt AHB with no past history of concurrent liver diseases, including hepatitis virus and human immunodeficiency virus infections or liver cirrhosis (13). In total, 118 patients with overt AHB were consecutively enrolled between January 2010 and December 2015. The diagnosis of overt AHB was made using the following criteria: (1) typical symptoms and signs of acute hepatitis; (2) serum alanine aminotransferase (ALT) levels ≥10 times the upper limit of normal and bilirubin levels ≥2 mg/dl; and (3) seropositivity for IgM antibody to HBV core (IgM anti-HBc), with a cutoff ratio ≥5.0 (13, 14). Hepatic decompensation was defined as a severe clinical syndrome with hepatic function impairment, as indicated by jaundice and a prolonged prothrombin time [PT; prolonged ≥3 s or an international normalized ratio (INR) ≥1.5] and/or occurrence of ascites/encephalopathy, as previously described (11, 13, 14).

All patients were treated with Nucs, including entecavir and tenofovir for various durations. After clinical recovery (normalization of PT and bilirubin), Nucs treatment was discontinued at the discretion of the physician. Liver biochemical and virological tests, as well as immunological assessments, were examined each week during the early stage of the disease, if indicated. The patients were then followed up every 1–6 months at the discretion of their physicians. Liver transplantation was performed for patients with model for end-stage liver disease (MELD) scores ≥35 or an initial MELD score < 35 that increased over the subsequent 1 to 2 weeks (15).

### Methods

The tests, including the serum levels of ALT, bilirubin, ammonia, PT, INR, albumin, creatine, estradiol, white blood cell count, and cell classification, were performed using routine automated techniques. Serum hepatitis B surface antigen (HBsAg), hepatitis B e antigen (HBeAg)/anti-HBe, anti-hepatitis D virus, IgM anti-hepatitis A virus (Abbott Diagnostics, North Chicago, IL, USA), IgM anti-hepatitis B core (Cobas, Roche Diagnostics, Pleasanton, CA, USA), and anti-hepatitis C virus antibodies (Axsym HCV, version 3, Abbott diagnostics, North Chicago, IL, USA) were assayed using commercial enzyme immunoassays. The HBV genotype was determined retrospectively using the polymerase chain reaction (PCR)-restriction fragment length polymorphism of the surface gene of HBV (16) (although not all tested patients’ sera typable for HBV genotypes). The serum levels of HBV DNA were assayed using an ultrasensitive PCR assay (Roche Diagnostics, Mannheim, Germany). Quantitative serum HBsAg (qHBsAg) (Roche Diagnostics), interferon-gamma-induced protein 10 (IP-10), soluble programmed cell death protein 1 (sPD-1), and Th1/Th2 immune assays, including interleukin (IL)-2, IL-4, IL-6, IL-10, IL-17A, interferon (IFN)-γ, and tumor necrosis factor (TNF) [Cytometric Bead Array (BD Biosciences, San Diego, CA, USA)], were assayed retrospectively using stored serum specimens. Given that it is a retrospective study, the stored sera were not available for every assay, and no intentional selection was done for any of the following assays. The qHBsAg, HBV genotype, HBeAg, estradiol, cytokines, and Th1/Th2 immune assays were assessed in 80, 48, 98, 20, 83, and 20 available stored sera, respectively. IHC studies were performed on the decompensated livers of AHB patients (n=3, none of the AHB patients received liver biopsy due to the risk of massive bleeding) and of CHB patients (n=5) who underwent liver transplantation. IHC analyses of cluster of differentiation 4 (CD4), CD8, CD15, CD56, PD-1, Interleukin 17 (IL-17), forkhead box P3 [FoxP3; for T regulatory cells (Treg cells)], and HBsAg were performed using anti-CD4 Ab, anti-CD8 Ab (Abcam, Inc. Cambridge, MA, USA), anti-CD15 Ab, anti-CD56 Ab (R&D Systems, MN, USA), anti-PD-1 Ab (Abcam, Inc. Cambridge, MA, USA), anti-IL-17 Ab, anti-FoxP3 Ab (R&D Systems, MN, USA), and anti-HBsAb (Virostat, Inc., Portland, ME, USA), respectively. Protein expression intensity was determined using ImageJ software (http://imagej.nih.gov/ij/, National Institutes of Health, USA). The data were tabulated and graphed using FlowCytomixPro 2.1 (Bender Medsystems, Vienna, Austria).

### Mice and Hydrodynamic Injection (HBT) of HBV Plasmid

A total of 13.5 μg of HBV1.3 plasmid was injected into the tail vein of 2- month-old (males, n=10; females, n=10) and 12-month-old (males, n=10; females, n=10) B6 mice in a volume of saline equivalent to 8% of the body mass of the mouse (17). The total volume was delivered within 5–8 s. Tail vain bleedings were performed every 24 h after HBT (at least four occasions). The serum levels of IL-17 (R&D Systems, MN, USA) were assayed using the tail vein serum.

### Statistics

All statistical analyses were performed using the Statistical Package for Social Sciences (SPSS version 21, SPSS Inc., Chicago, USA). For between-group comparisons, continuous variables were analyzed using Student’s *t*-test or non-parametric Mann-Whitney U tests, whereas categorical variables were analyzed using a chi-squared test or the Fisher exact test, where appropriate. Continuous variables were summarized as the means +/− standard deviations (SDs) and medians (ranges); categorical variables were summarized as numbers and percentages (%). Multivariate linear regression models were used to assess the relationship between various dependent and independent variables by adjusting for all independent variables with a *p*-value of <0.1 in the univariate analyses. The co-linearity of the different variables was determined using linear regression tests. Statistical significance was defined as the 5% level based on a two-tailed test of the null hypothesis.

### Institutional Review Board

The study protocols for human and animal studies conformed to the ethical guidelines of the 1975 Declaration of Helsinki and were approved by the hospital’s institutional review board and Institutional Animal Care and Use Committee.

## Results

### Baseline Characteristics

The baseline characteristics of the 118 patients (females, 47.5%) and the comparisons between the compensated and decompensated cases are shown in **Table 1**. Of the 41 patients with decompensated AHB, 32 (78.1%) were female and 20 (48.7%) had baseline hepatic encephalopathy; 8 patients (3 females) died between 1 day and 2 months after admission, and 3 (2 females) underwent liver transplantation between 4 and 9 days after admission. Compared with the compensated patients, the patients with decompensated AHB were more often female, older, and had higher levels of ALT, bilirubin, INR, WBC counts, and a higher neutrophil-lymphocyte ratio (NLR); however, they had a lower prevalence of genotype C of HBV infection and HBeAg positivity as well as platelet counts and qHBsAg.

**Table 1:** Baseline characteristics and comparisons between overt acute hepatitis B (AHB) patients with and without hepatic decompensation.

	Total AHB	Decompensation		*p* values
	N=118	Yes (n=41)	No (n=77)	
	Median/mean ± standard deviation (range)			
Female^*^	56 (47.5)	32 (78.1)	24 (31.2)	<0.001
Age (year)	38.0/40.2 ± 13.5 (18~83)	49.0/47.0 ± 13.8 (19~74)	34.5/36.6 ± 11.8 (18~83)	<0.001
ALT (U/L)	2,208/2,446 ± 1,421 (411~7,110)	3,224/3,495 ± 1,708 (879~7,110)	1,846/1,880 ± 801 (411~3,857)	<0.001
Bilirubin (mg/dl)	8.25/9.22 ± 5.61 (2.0~28.5)	10.9/12.07 ± 5.88 (3.0~24.5)	7.2/7.67 ± 4.82 (2.0~28.5)	0.001
INR	1.31/2.11 ± 2.11 (1.0~12.0)	2.3/3.67± 2.84 (1.5~12.0)	1.2/1.18 ± 0.13 (1.0~1.4)	<0.001
NLR	2.36/2.96 ± 1.85 (0.71~12.51)	3.26/3.74 ± 2.47 (0.79~12.57)	2.15/2.49 ± 1.13 (0.71~5.46)	0.004
Platelet counts (10^3^/ul)	188.5/200.8 ± 81.15 (64~534)	146.0/151.7 ± 59.40 (64~397)	225.0/229.9 ± 78.52 (65~534)	<0.001
Ammonia (μg/dl)	95.0/116.8 ± 66.54 (31~304)	144.0/154.5 ± 67.72 (63~304)	73.5/72.78 ± 26.30 (31~135)	<0.001
Genotype C^*#^ (n=48)	9 (18.7)	0 (0)	9 (33.3)	<0.001
HBeAg-positive^*^ (n=98)	34 (34.7)	4 (12.1)	30 (46.2)	<0.001
HBV DNA (log_10_IU/ml)	4.65/4.57 ± 1.38 (1.54~8.48)	4.11/4.41 ± 1.34 (2.55~8.48)	4.88/4.68 ± 1.41 (1.54~7.79)	0.377
HBsAg (log_10_IU/ml)	2.10/1.94 ± 1.64 (-0.99~5.36)	0.53/1.04 ± 1.60 (-0.99~5.36)	3.06/2.55 ± 1.38 (-0.92~4.57)	<0.001

*n (%); ALT, alanine transaminase; INR, international normalized ratio; NLR, neutrophil-to-lymphocyte ratio; #The other genotype is genotype B.

### Sex, Age, and ALT Levels Were Independently Associated With Hepatic Decompensation

The univariate analyses showed that sex, age, HBeAg positivity, and ALT and qHBsAg levels were associated with hepatic decompensation, and the multivariate analyses confirmed that sex, age, and ALT levels were independently associated with hepatic decompensation (**Table 2**).

**Table 2:** Baseline factors associated with hepatic decompensation in the patients with overt acute hepatitis B.

Baseline factors	Univariate analyses		Multivariate analyses	
	OD (95% CI OD)	*p* values	OD (95% CI OD)	*p* values
Sex (female)	7.85 (3.25~18.98)	<0.001	7.68 (1.07~54.9)	0.042
Age (year)	1.06 (1.025~1.094)	0.001	1.22 (1.06~1.40)	0.006
ALT (U/L)	1.00 (1.001~1.002)	<0.001	1.002 (1.001~1.004)	0.003
HBeAg-positive	0.16 (0.051~0.51)	0.002	0.232 (0.026~2.09)	0.193
HBV DNA (log_10_IU/ml)	0.87 (0.636~1.197)	0.398		
HBV genotype (genotype B)	0.294 (0.259~1.847)	0.999		
HBsAg (log_10_IU/ml)	0.52 (0.375~0.732)	<0.001	0.631 (0.31~1.27)	0.196

OD, odds ratio; 95% CI OD, 95% confidence interval of odds ratio; ALT, alanine transaminase.

### Higher sPD-1 Levels but Lower IP-10, IL-4, and IFNγ Levels Were Noted in the Decompensated Patients Compared With Those in the Compensated Patients

**Figure 1:**
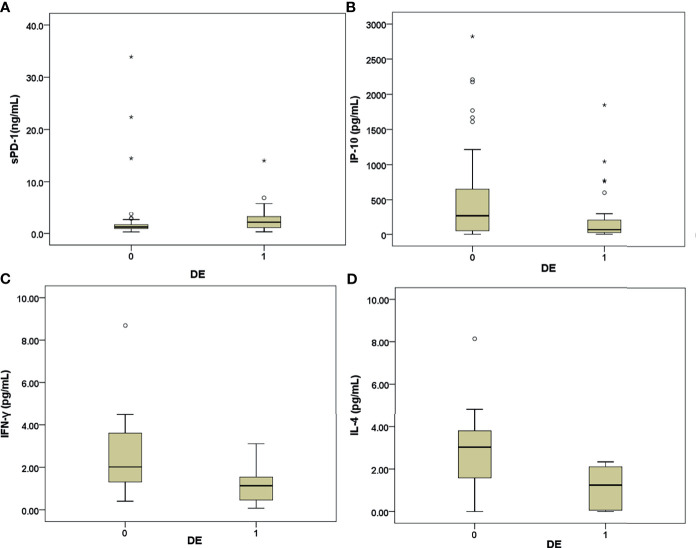
**(A–D)** Box-and-whisker plots of the baseline levels of sPD-1 **(A)**, IFN-γ-IP-10 **(B)**, INF-γ **(C)**, and IL-4 **(D)** in patients with decompensated (DE:1) and compensated (DE:0) AHB. The outliers are presented as circles or stars.

The patients with decompensated AHB had higher levels of sPD-1 but lower levels of IP-10, IFN-γ, and IL-4 than the compensated AHB patients (**Table 3** and **Figure 1**). However, the estradiol levels of patients with and without decompensation were not significantly different (**Table 3**).

**Table 3:** Comparisons of the differences of hormone, cytokine, and immune response between overt acute hepatitis B (AHB) patients with and without hepatic decompensation.

	Total AHB	Decompensation		*p* values
	Median (Range)			
		Yes	No	
**Estradiol** (pg/ml)	77.98 (5~444)	79.65 (5~270)	77.99 (40~444)	0.904
**Cytokines**				
IP-10 (pg/ml)	167.9 (0.764~2208)	68.03 (0.764~1847)	257.3 (1.537~2208)	0.036
sPD-1 (ng/ml)	1.37 (0.30~4.47)	2.15 (0.31~1.4)	1.21 (0.30~4.4)	0.017
**Th1/Th2 immune response**				
IL-2 (pg/ml)	0 (0~10.0)	0 (0~1)	0 (0~10.0)	0.152
IL-4 (pg/ml)	1.984 (0~8.140)	1.238 (0~2.330)	3.028 (0~8.140)	0.044
IL-6 (pg/ml)	7.460 (0.889~86.43)	6.804 (1.640~86.43)	7.460 (0.889~46.54)	0.487
IL-10 (pg/ml)	3.085 (0~28.37)	1.817 (0~5.257)	4.408 (0~28.37)	0.113
IL-17A (pg/ml)	32.91 (0~92.67)	25.39 (0~74.95)	56.92 (9.286~92.67)	0.27
IFN-γ (pg/ml)	1.541 (0.076~8.687)	1.137 (0.076~3.108)	2.014 (0.403~8.687)	0.049
TNF (pg/ml)	2.263 (0~11.35)	2.474 (0~4.407)	2.263 (0~11.35)	0.62

IP-10, interferon-γ-inducible protein 10; sPD-1, soluble programmed cell death protein 1; Th1, T helper 1 cell; Th2, T helper 2 cell; IL-2, interleukin-2; IL-4, interleukin-4; IL-6, interleukin-6; IL-10, interleukin-10; IL-17A, interleukin-17A; IFN-γ, interferon-γ; TNF, tumor necrosis factor.

### Higher sPD-1 but Lower IL-4 and IFNγ Levels Were Observed in the Female Patients Compared With Those in the Male Patients

**Figure 2:**
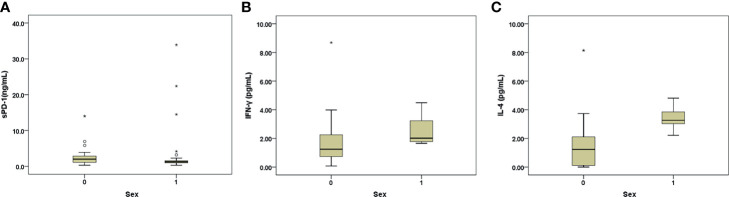
**(A–C)** Box-and-whisker plots of the baseline sPD-1 **(A)**, INF-γ **(B)**, and IL-4 **(C)** levels in males (sex: 1) and females (sex: 0) with AHB. The outliers are presented as circles or stars.

Female patients showed higher levels of INR and sPD-1 but lower levels of qHBsAg, IL4, and IFN-γ and lower prevalence of HBeAg positivity than the male patients. The females had borderline higher levels of ALT and lower rate of genotype C HBV infection than the males (**Table 4** and **Figure 2**). In addition, the females ≥50 yrs (the average age of menopause for most Taiwanese females) (n=30) (18) had lower platelet levels and rates of genotype C HBV infection but higher levels of INR and ammonia (**Table 4**) and prevalence of decompensation (63.3 *vs.* 15%, *p*<0.001) than those <50 yrs (n=88).

**Table 4:** Comparisons of the differences of baseline age, biochemistry, hormone, cytokine, and immune response between male and female as well as old and young overt acute hepatitis B patients.

	Males	Females	*p* values	Young (≤50 yrs)	Old (>50 yrs)	*p* values
**Case number**	62	56		77	41	
**Sex** [male, (%)]	100	0		54.5	46.7	0.46
	Median (Range)	Median (Range)		Median (Range)	Median (Range)	
**Age** (year)	37.0 (18~83)	38.5 (18~74)	0.326	34.0 (18~50)	55.5 (51~83)	<0.001
**Biochemistry**						
ALT (U/L)	2159 (411~6465)	2360 (446~7110)	0.091	2234 (411~7110)	2135 (442~6274)	0.521
Bilirubin (mg/dl)	7.45 (2.0~28.5)	9.15 (2.0~23.0)	0.527	7.7 (2.0~28.5)	9.3 (2.0~22.3)	0.125
INR	1.2 (1.0~12.0)	1.65 (1.0~12.0)	<0.001	1.2 (1.0~12.0)	1.8 (1.0~12.0)	0.002
NLR	2.3 (1.12~7.8)	2.44 (0.71~12.57)	0.672	2.2 (0.71~12.51)	2.8 (1.26~10.19)	0.226
Platelet counts (10^3^/ul)	206 (65~534)	179.5 (64~397)	0.135	205 (65~534)	152 (64~341)	0.004
Ammonia (μg/dl)	95 (43~304)	90.0 (31~228)	0.292	82 (31~236)	121 (63~304)	0.024
Genotype C (%)	25	11.5	0.099	25.7	0	0.008
HBeAg-positive (%)	50	17.4	<0.001	38.9	23.1	0.128
HBVDNA (log_10_IU/ml)	5.0 (1.5~7.2)	4.11 (2.3~8.5)	0.088	4.40 (1.54~7.79)	4.82 (2.7~8.5)	0.102
HBsAg(log_10_IU/ml)	2.53 (−0.5~4.6)	0.79 (−0.9~5.3)	0.007	2.45 (−0.9~5.36)	2.10 (−0.7~3.88)	0.361
**Estradiol** (pg/ml)	67.7 (52~98)	81.3 (5~444)	0.5	77.9 (40~444)	68.13 (5~266)	0.416
**Cytokines**						
IP-10 (pg/ml)	185.2 (2~1769)	150.4 (1~2823)	0.888	145.5 (2~2208)	203.9 (1~2823)	0.751
sPD-1 (ng/ml)	1.24 (0.30~33.8)	2.00 (0.31~44.7)	0.023	1.37 (0.30~44.7)	1.32 (0.31~14.0)	0.977
**Th1/Th2 immunity**						
IL-2 (pg/ml)	0.78 (0~4)	0 (0~10)	0.186	0 (0~10)	0 (0~4)	0.99
IL-4 (pg/ml)	3.26 (2.21~4.80)	1.23 (0~8.14)	0.014	2.09 (0~8.14)	1.58 (0~3.26)	0.282
IL-6 (pg/ml)	10.8 (1.53~26.09)	4.77 (0.88~86.4)	0.823	6.2 (0.88~86.4)	11.5 (1.64~20.5)	0.831
IL-10 (pg/ml)	3.44 (0~10.6)	2.00 (0~28.3)	0.823	2.60 (0~28.3)	3.74 (0~12.7)	0.765
IL-17A (pg/ml)	61.8 (9.28~92.6)	25.3 (0~74.9)	0.107	21.2 (0~92.6)	37.2 (1.60~61.8)	0.966
IFN-γ (pg/ml)	2.01 (1.65~4.49)	1.25 (0.075~8.68)	0.048	1.77 (0.18~8.68)	1.48 (0.07~3.23)	0.467
TNF (pg/ml)	3.53 (2.26~4.26)	1.51 (0~11.35)	0.219	2.82 (0~11.35)	0.70 (0~4.11)	0.106

IP-10, interferon-γ-inducible protein 10; sPD-1, soluble programmed cell death protein 1; Th1, T helper 1 cell; Th2, T helper 2 cell; IL-2, interleukin-2; IL-4, interleukin-4; IL-6, interleukin-6; IL-10, interleukin-10; IL-17A, interleukin-17A; IFN-γ, interferon-γ; TNF, tumor necrosis factor.

### IL-17-Positive Cell Predominance With Low CD4/CD8 Ratios and Few HBsAg-Positive Cells in the Decompensated AHB Livers

**Figure 3:**
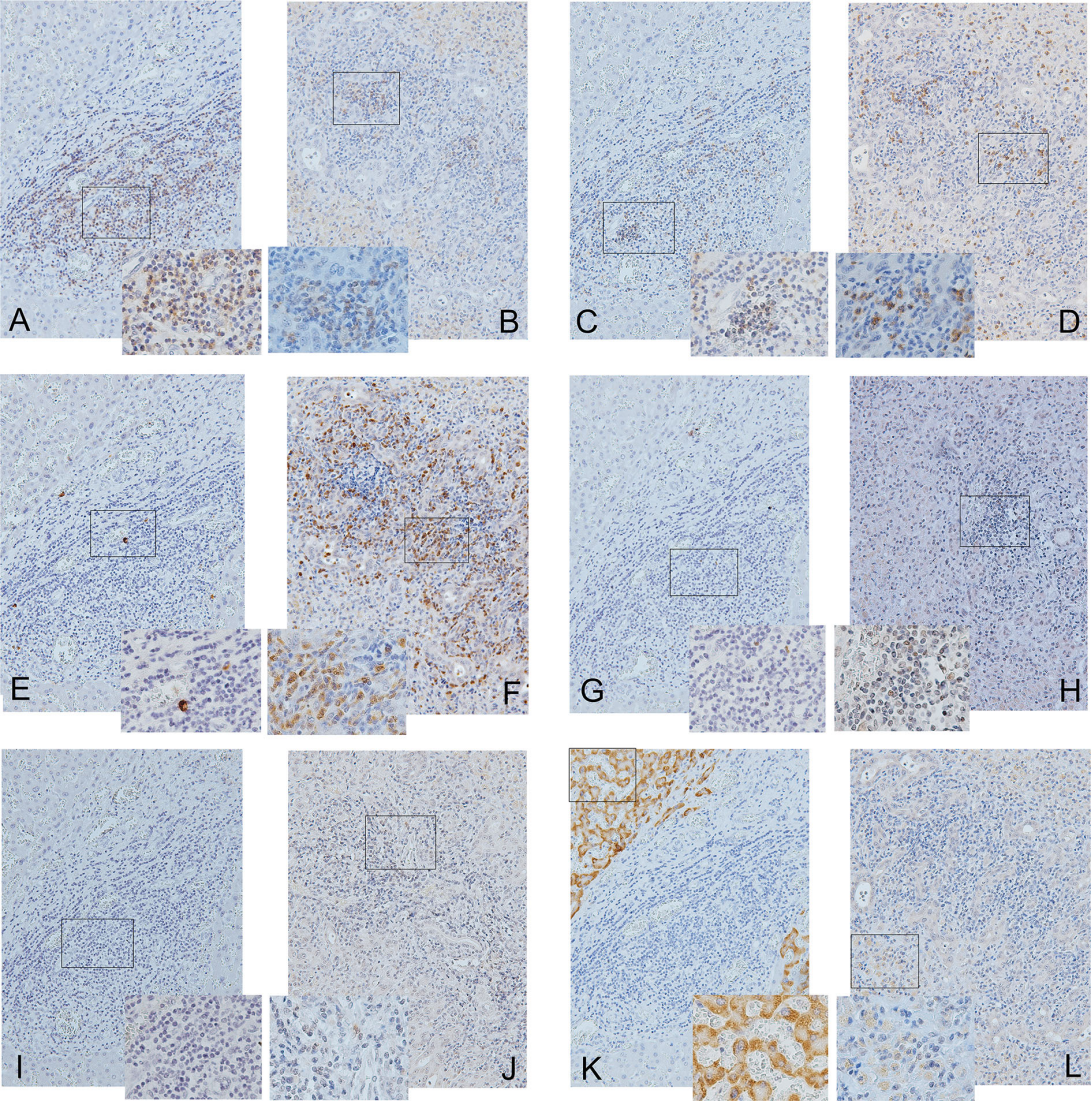
Immunohistochemical studies of CD4 **(A, B)**, CD8 **(C, D)**, IL-17 **(E, F)**, Treg **(G, H)**, PD-1 **(I, J)**, and HBsAg and **(K, L)** in the decompensated livers of a male patient with chronic hepatitis B **(A, C, E, G, I, K)** and a female patient with acute hepatitis B **(B, D, F, H, J, L)**. The panels are shown at 200× magnification. The frames inside the panels are the focused regions enlarged at 400× magnification (the right or left lower quadrant of each panel). Positive cells are stained brown.

**Figure 3** shows the IHCs of the representative case of decompensated male CHB (**Figures 3A, C, E, G, I, K** from a male patient) and female AHB (**Figures 3B, D, F, H, J, L** from a female patient). For AHB, generally, most of the liver was invaded by extensive inflammatory cells that encroached on the remaining hepatocytes (solitary or in “islands”). Specifically, CD8-positive cells (**Figure 3D**) scattered more diffusely and extensively than CD4-positive cells, which tended to form clusters (**Figure 3B**). The IL-17-positive cells (**Figure 3F**) were distributed extensively and behaved as the predominant inflammatory cells in the decompensated AHB livers. Many of the IL-17-positive cells were Th17 cells and neutrophils, which simultaneously expressed CD4 (**Figures 3B, F**) and CD15 (**Supplementary Figure 1**), respectively. In contrast, negligible Treg (**Figure 3H**), PD-1-positive cells (**Figure 3J**), and NK cells (CD56+ cells) were identified. In addition, some residual hepatocytes weakly expressed HBsAg (**Figure 3L**).

Compared with decompensated livers of CHB, more IL-17-positive cells [41.9+/−13.5 *vs.* 0.12+/−0.05% (denominators: non-parenchymal cells), *p*=0.001, **Figures 3E, F**] and Th17 cells (18.88+/−10.57 *vs.* 0.12+/−0.05%, *p*=0.016, **Figures 3A, B, E, F**) but fewer HBsAg-positive hepatocytes (23.12 +/−14.68 *vs.* 62.97 +/− 13.56%, *p*=0.001, **Figures 3K, L**) and lower CD4/CD8 ratios (0.88 +/−0.22 *vs.* 3.2+/−1.6, *p*=0.032, **Figures 3A–D**) were observed in the decompensated livers of AHB. Both the Treg and PD-1-positive cells were scarce in both AHB and CHB (**Figures 3G–J**).

### Higher Serum IL-17 Levels in the Female Mice Than Those in the Male AHB Mice

**Figure 4:**
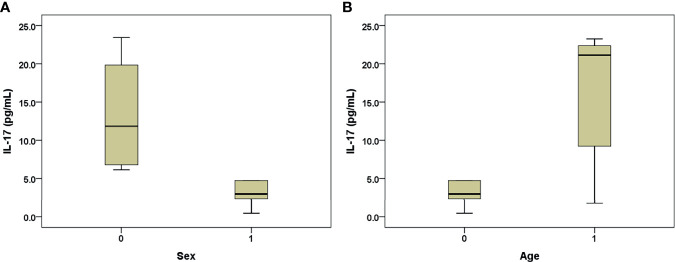
Box-and-whisker plots of the 24-h post-transfection serum IL-17 levels in the B6 mice that underwent hydrodynamic injection of HBV plasmid. **(A)** Young mice, males (sex: 1) and females (sex: 0); **(B)** male mice, old (age: 1) and young (age: 0). The outliers are presented as circles or stars.

Among the 2-month-old B6 mice, 24 h after HBT with PHBV1.3, the female mice had higher IL-17 levels than the male mice (13.31+/−8.09 *vs.* 3.05+/−1.62 pg/ml, *p*=0.01) (**Figure 4A**). However, among the 12-month-old mice, no difference was observed in the IL-17 levels between the male and female mice (*p*=0.527). When we stratified the mice by sex, the old mice had higher IL-17 levels than the young mice only in the male subgroup (23.79+/−19.10 *vs.* 3.05+/−1.62 pg/ml, *p*=0.022) (**Figure 4B**).

## Discussion

That the decompensated AHB patients had higher ALT (reflecting the hepatic immune response) (19) and NLR levels (reflecting the general immune response) (20) but lower qHBsAg levels and HBeAg positivity (21) than the compensated cases indicates a more severe battle between the virus and host immunity, with more efficient HBV clearance in the former. Notably, females predominated among the decompensated AHB cases and showed borderline higher ALT levels but lower qHBsAg and HBeAg positivity than the males. On the other hand, PD-1 and its ligands (PD-L) are responsible for the inhibitory T-cell signaling (22), while sPD-1 is encoded by one of the alternatively spliced PD-1 mRNA transcripts, promoting T-cell responses by blocking the PD-1/PD-L pathway (23). The decompensated AHB patients exhibited higher serum sPD-1 levels than the compensated patients, and negligible hepatic PD-1-positive cells were observed. Also, the female patients showed higher sPD-1 levels than the males. These observations suggest a stronger T-cell immune response in the decompensated and female AHB than their counterparts, and that the underlying immunology in severe AHB is likely associated with sexual dimorphism. It is consistent with the notions that women exhibit more robust immune responses than men to clear the virus (24), and men predominate in HBV carriers (25). In addition to sex, age and ALT levels were associated with decompensation. Aging is a poor AHB prognostic factor (26) because elderly patients usually have higher bilirubin but lower ALT levels (27) than young patients. Interestingly, the decompensated patients were older but had higher ALT levels than the compensated cases, and no differences in the ALT levels were observed between those >50 yrs and those ≤50 yrs. Thus, aging alone cannot fully account for decompensation; rather, it may have a synergistic effect with sex and contribute to decompensation in AHB.

Importantly, many Th17 cells, an independent subset of CD4 cells characterized by the production of their signature cytokine IL-17 (28), were found in the decompensated AHB livers, in contrast to scarce Th17 cells in the decompensated CHB livers. Th17 cells are the primary cells involved in autoimmunity and destructive immune diseases (29), while autoimmune diseases are characterized by a significant female bias (30). Furthermore, the development of Th17 cells is suppressed by IFNγ and IL-4, which promote Th1 and Th2 cells, respectively (31), and Th17 responses emerge as an early reaction to numerous pathogens not controlled by Th1- or Th2-type immunity (29). Although the investigated estradiol data failed to provide helpful information regarding sex-dimorphic immunity, the finding that female and decompensated AHB patients showed lower levels of IFN-γ and IL-4 than male and compensated AHB patients, respectively, indicates that the acceleration of Th17 cell development may be associated with the sex-dimorphic immunity. Interestingly, the representing IHC cases for the decompensated AHB and CHB patients were female and male, respectively, echoing the sex ratios for the severe forms of AHB (13) and CHB (11, 12, 25). The higher hepatic Th17 ratios in the decompensated AHB cases (compared to CHB) aligned with a more potent destructive immunity in AHB and likely reflected the sex-dimorphic immune intensity to HBV infection. The serum IL-17 levels did not differ either between the decompensated and compensated or between the female and male AHB patients, thus, measurement of serum IL-17 might be of limited clinical use and cytokines other than IL-17 might also play some roles in decompensated AHB. Anyhow, our AHB animal model confirmed the early serum surge of IL17 in the female mice, indicating that the IL-17-associated pathway might initiate holistically in an early stage of AHB before being blunted by complicated immune cascades while patients seek medical help. Consistently, in *de novo* HBV-infected patients after orthotopic liver transplantation, the viral clearance was accompanied by a rapid increase in serum Th17 cells during the first month (32). By contrast, Treg cells downregulate the immunity, including the differentiation and activity of Th17 cells (33), and they were extremely scarce in the decompensated livers. However, Th17 cells are not the only source of IL-17; innate immune cells can produce IL-17 (28). Consistent with previous study (34), both Th17 cells and neutrophils constituted the IL-17-positive cells in decompensated AHB livers. Given the broad distribution of IL-17 receptors in liver cells (28, 35), IL-17 is the key cytokine in the recruitment and activation of neutrophils and monocytes (36), and IL-17 was reported to suppress HBV replication (37), all IL-17-positive cells might participate in the immune cascade to induce a massive tissue reaction in expelling HBV. Because aging promotes neutrophil-induced mortality by augmenting IL-17 production during viral infection in mice (38), there may be a connection between aging and destructive immunity-related diseases through IL-17-associated pathways. The higher serum IL-17 levels observed in the old male AHB mice compared to the young supported the crucial role that IL-17 plays in the age-aggravated severity of AHB. In contrast, among the female mice, the immunity associated with estrogen (24) in the young mice and with aging in the old mice (38), both IL-17-mediated, might mute the difference of serum IL-17 between the young and old mice.

Inflammatory cells are dependent on IFN-γ to secrete IP-10 (36). The IP-10 levels are a marker of Th1-oriented T-cell immune response (39). In CHB patients, IP-10 is produced by hepatocytes in inflammatory areas (40), and higher pretreatment IP-10 levels are associated with a favorable response to INF- (41) and Nuc-based therapies (40). Paradoxically, lower baseline IP-10 levels were observed in the decompensated than in the compensated AHB patients, despite the extensive immune responses. This result might be due to the lower IFN-γ levels associated with relatively low Th1 immunity to enhance IP-10 production (31).

The CD4/CD8 ratios of liver-derived lymphocytes are positively correlated with serum HBV DNA levels (42), which predict hepatic decompensation in CHB (43). Moreover, the serum viral load of AHB is significantly lower than that of CHB (7), and HBsAg-positive hepatocytes were lower in the AHB than the CHB cases with decompensation. Thus, the lower CD4/CD8 ratios in the livers of the AHB cases compared to the CHB cases might be associated with a lower viral load in the former, at the time of decompensation.

In line with the finding that genotype C HBV infection is a risk factor for chronicity (44) and that genotype B HBV infection accounts for most fulminant AHB cases (26), elderly patients with decompensated AHB had fewer genotype C (i.e., more genotype B) HBV infections than their counterparts, and the females had borderline fewer genotype C HBV infection than the males. Whether genotype B HBV infection tends to elicit a more severe immune reaction in the aged and females requires further investigation.

The current study has several limitations. First, although the route of HBV infection might affect HBsAg clearance, we cannot confirm the role of the transmission route in the development of decompensated AHB, as a definitive history of exposure to HBV cannot be traced in most (up to 80%) cases (45). Second, because of the low viral titer in decompensated cases, the effects of viral mutation (26) on decompensation cannot be thoroughly assessed. Third, because of the retrospective nature of this study, a definite causal relationship among the various immune reactions cannot be confirmed. Fourth, all patients had been prescribed Nucs starting at the early stage of admission. Whether Nucs affected the natural course of the AHB is unclear. Future prospective studies of AHB with comprehensive examinations of transmission routes, virological factors, cellular immunological tests, and a uniform antiviral regimen are required to verify the precise role that destructive immunity plays in patients with decompensated AHB.

**Figure 5:**
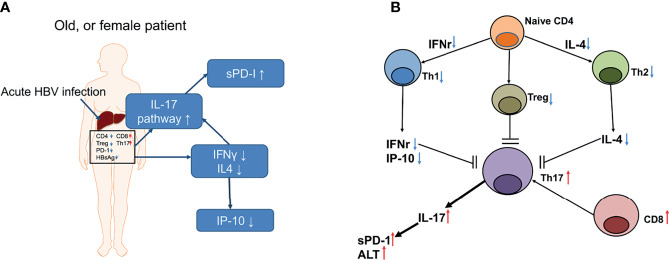
A summary of the events that occurred in a representative old, or female patient with decompensated AHB **(A)** and the proposed T cell interactions in the interleukin 17 (IL-17)-centered, hepatic destructive pathway **(B)**. **(A)** The notes in the blue frames depict the upregulated (upward arrows) or downregulated (downward arrows) cytokines or pathway. sPD-1, soluble programmed death-1; IL-17, interleukin 17; INF-γ, interferon gamma; IL4, interleukin-4; IP-10, interferon gamma-induced protein 10. Words in the frame depicted the hepatic IHC results; CD4, cluster of differentiation 4; CD8, cluster of differentiation 8; PD-1, programmed cell death protein 1; Th17, T helper 17 cell; Treg, regulatory T cell; HBsAg, hepatitis B surface antigen. Small red arrows: upregulation; small blue arrows: downregulation. **(B)** Black arrow heads: stimulation; black blunt ends: inhibition; small red arrows: upregulation; small blue arrows: downregulation.

Overall, destructive immunity might be crucial in female sex-related overt decompensated AHB. The proposed scenario is shown in **Figure 5**. Briefly, destructive immunity may be augmented by downregulating Th1 and Th2 immune responses and by accelerating IL-17-associated pathway, which is a potential therapeutic target to prevent fatality or liver transplantation among patients with decompensated AHB.

## Data Availability Statement

The original contributions presented in the study are included in the article/**Supplementary Material**. Further inquiries can be directed to the corresponding author.

## Ethics Statement

The studies involving human participants were reviewed and approved by Chang Gung Memorial Hospital. The ethics committee waived the requirement of written informed consent for participation.

## Author Contributions

C-TY and R-NC: statistical analysis and manuscript writing. Y-FL: data collection, manuscript writing and critical revision of the manuscript for important intellectual content. M-LC: study design and implementation, manuscript drafting, and critical revision of the manuscript for important intellectual content. All authors contributed to the article and approved the submitted version.

## Funding

This study was supported by grants from the Chang Gung Medical Research Program (CMRPG3I0412, CMRPG3K0721, CMRPG1K0111, and CMRPG1K0112), the National Science Council, Taiwan (MOST 109-2314-B-182-024-, 109-2629-B-182-002-, 110-2629-B-182-001-, and 110-2314-B-182-044-), and the Prosperous Foundation, Taipei, Taiwan. The funders had no role in study design, data collection and analysis, decision to publish, or preparation of the manuscript.

## Conflict of Interest

The authors declare that the research was conducted in the absence of any commercial or financial relationships that could be construed as a potential conflict of interest.

## Publisher’s Note

All claims expressed in this article are solely those of the authors and do not necessarily represent those of their affiliated organizations, or those of the publisher, the editors and the reviewers. Any product that may be evaluated in this article, or claim that may be made by its manufacturer, is not guaranteed or endorsed by the publisher.

## Supplementary Material

Supplementary Figure 1 Immunohistochemical studies of IL-17 **(A)** and CD15 **(B)** (200×) in the decompensated livers of a female patient with acute hepatitis B. The positive cells are stained in brown.
