# Supplementary figures and images for: Overt Acute Hepatitis B Deteriorates in Females: Destructive Immunity With an Exaggerated Interleukin-17 Pathway
Source: Front Immunol. 2021 Nov 11;12:631976. doi: 10.3389/fimmu.2021.631976 (PMC8631789; doi:10.3389/fimmu.2021.631976)

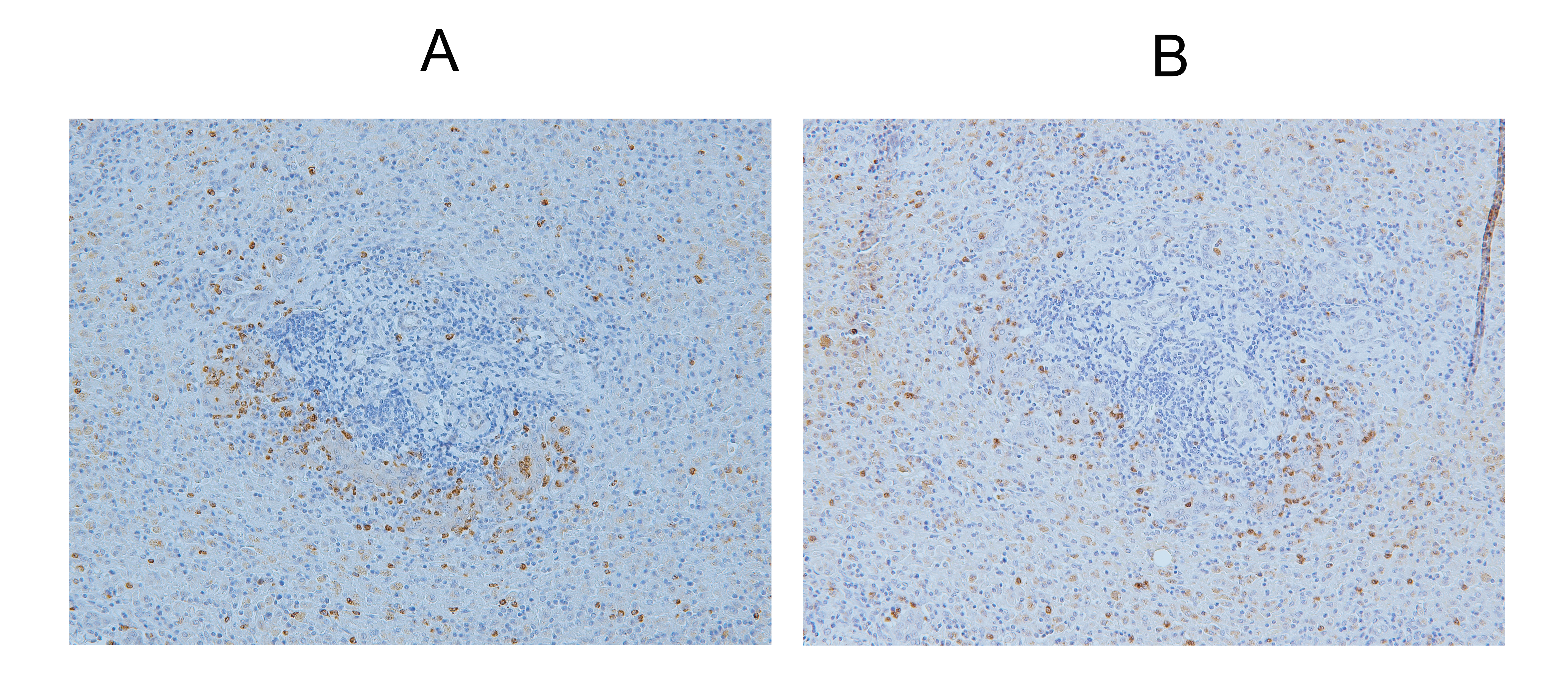

Supplement: Supplementary Figure 1 — Immunohistochemical studies of IL-17 (A) and CD15 (B) (200×) in the decompensated livers of a female patient with acute hepatitis B. The positive cells are stained in brown. [file Image_1.tif]
